# Efficacy and safety of remimazolam for sedation in gastrointestinal endoscopy: a systematic review and meta-analysis

**DOI:** 10.3389/fmed.2026.1811767

**Published:** 2026-06-24

**Authors:** Yuxiao Kong, Anqi Wang, Wenxia Jia

**Affiliations:** 1 School of Anesthesiology, Shanxi Medical University, Taiyuan, Shanxi, China; 2 Department of Anesthesiology, The First Hospital of Shanxi Medical University, Taiyuan, Shanxi, China

**Keywords:** benzodiazepine, efficacy, endoscopic sedation, gastrointestinal endoscopy, remimazolam

## Abstract

**Background:**

Gastrointestinal endoscopy serves as a key tool for diagnosing and treating digestive disorders. Adequate sedation is indispensable in achieving ideal surgical outcomes and promoting patient comfort. Recently, remimazolam has been utilized more frequently because of its good safety features, brief action time and rapid onset. This study aims to systematically review and meta-analyze remimazolam’s effectiveness and safety profile in sedating patients undergoing gastrointestinal endoscopies.

**Methods:**

We conducted literature searches in Embase, PubMed, and the Cochrane Library databases, covering the period from the establishment of each database to January 31, 2026 for all relevant CCTs and RCTs that discussed remimazolam sedation for gastrointestinal endoscopy. RevMan software (version 5.4) was employed for all statistical analyses.

**Results:**

Eight trials involving 2,455 patients were included. In cardiovascular outcomes, remimazolam was linked to a significantly lower hypotension (RR = 0.59; 95% CI: 0.43–0.81) and bradycardia (RR = 0.42; 95% CI: 0.25–0.71) risks compared with midazolam and propofol. For respiratory adverse events, remimazolam demonstrated notably reduced rates of apnea (RR = 0.51; 95% CI: 0.30–0.88), oxygen desaturation (RR = 0.20; 95% CI: 0.08–0.52) and diminished respiratory compromise (RR = 0.45; 95% CI: 0.23–0.87). Regarding patient comfort, remimazolam significantly reduced injection-related pain (RR = 0.20; 95% CI: 0.06–0.66).

**Conclusion:**

Remimazolam may confer a favorable safety profile for procedural sedation in adults receiving gastrointestinal endoscopies. It could serve as a reliable and effective alternative to conventional sedatives.

## 1 Introduction

Numerous endoscopic procedures dedicated to the gastrointestinal (GI) system, including endoscopic retrograde cholangiopancreatography (ERCP), esophagogastroduodenoscopy (EGD), and colonoscopy, are widely employed for the diagnosis and management of digestive tract disorders. Sedation during these procedures can improve the patient’s experience and help the endoscopists perform the endoscopic procedure successfully (1, 2). There are many differences in the methods of sedation, and the sedative drugs are diverse from each other, too. Emerging research evidence suggests that remimazolam may hold distinct edges over currently available sedatives (3).

Remimazolam, an innovative ultra-short-acting benzodiazepine derivative, mediates sedative and anesthetic responses by positively allosterically modulating *γ*-aminobutyric acid type A (GABA_A_) receptors. Unlike many sedatives, it does not rely on hepatic or renal metabolism; instead, it is rapidly hydrolyzed by tissue cholinesterases. This extrahepatic metabolic pathway ensures stable pharmacokinetics, minimal drug accumulation, and a low risk of prolonged sedation, even in patients with mild organ dysfunction. In addition, its sedative effect has a rapid onset, short duration, and high predictability, and can be completely and rapidly reversed by the specific antagonist flumazenil, which is an antagonist of the positive allosteric modulator effects of benzodiazepines. This unique pharmacokinetic profile contributes to its favorable safety profile and lower incidence of side effects like bradycardia and respiratory depression (4, 5). Evidence also suggested that remimazolam demonstrated efficacy in Phase III trials conducted for patients who required endoscopies. Compared to currently available sedatives, remimazolam significantly reduced the sedation time and recovery time, which is beneficial for improving clinical efficiency (6).

Despite these promising findings, additional researches are required to clarify its clinical advantages as well as long-term safety. In view of this, we designed and implemented a meta-analysis to comprehensively evaluate remimazolam’s safety and effectiveness as a sedative agent during GI endoscopic interventions. Our analysis concentrated on time variables, adverse events which are categorized by system (cardiovascular events, respiratory events, injection site pain and neurological symptoms) and sedation quality.

## 2 Methods

**Figure 1:**
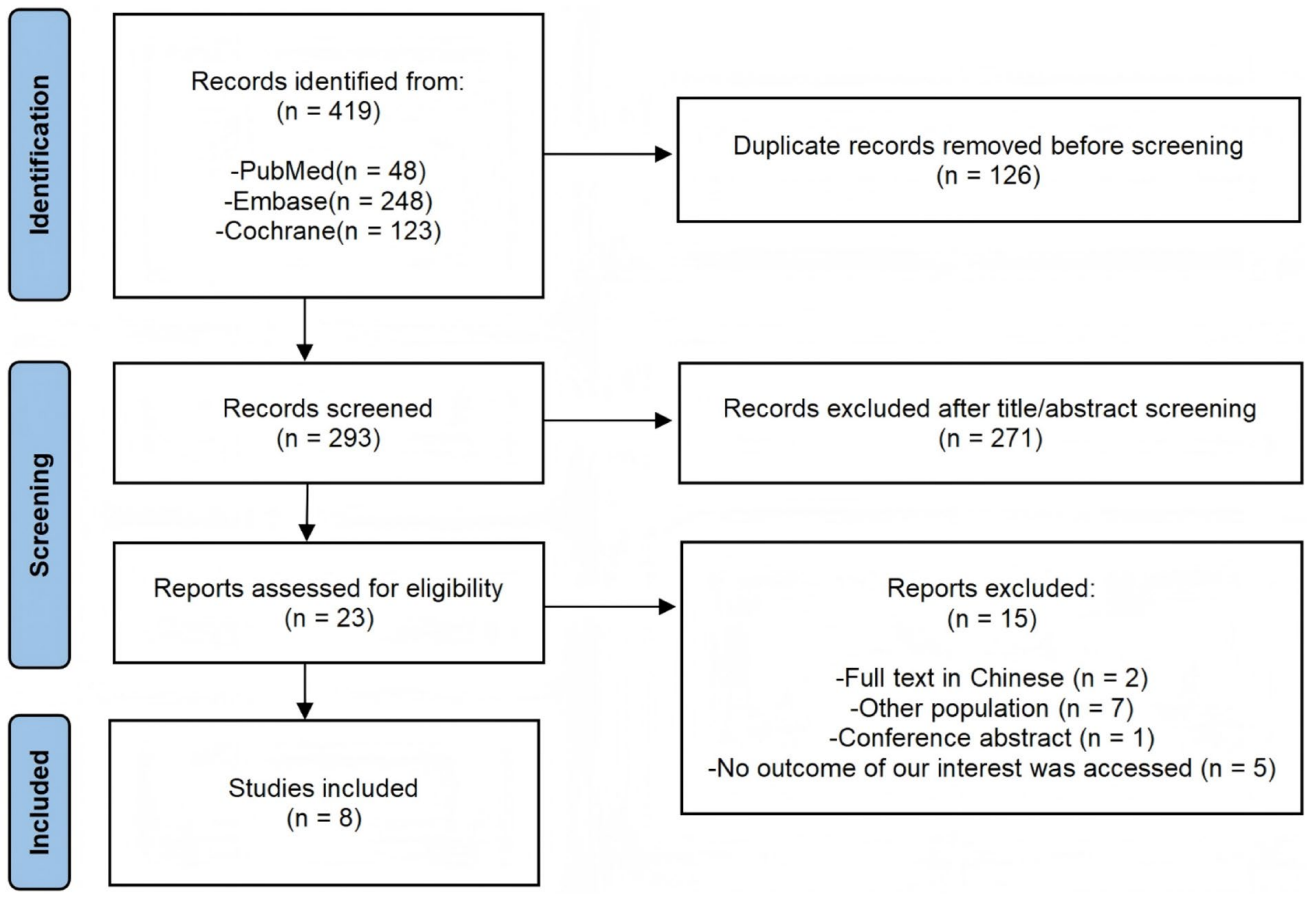
Flowchart outlining study identification, screening, and inclusion.

All analytical procedures in this study followed the PRISMA 2020 reporting standards (7). The protocol for the present study was registered in PROSPERO with the registration identifier CRD420261302876. The workflow for literature identification and selection is depicted in Figure 1.

### 2.1 Search protocol

On January 31, 2026, we comprehensively searched Embase, PubMed, and the Cochrane Library to obtain all pertinent controlled clinical trials (CCTs) and randomized controlled trials (RCTs). Search strategies were formulated combining both MeSH terms and associated free-text keywords, involving “remimazolam,” “CNS-7056,” “gastrointestinal endoscopy” and “endoscopic gastrointestinal surgery.” Supplementary Table 1 contains the complete, predefined search syntax per database. Given that this is a meta-analysis, neither institutional review board approval nor ethical committee authorization was deemed necessary.

### 2.2 Eligibility requirements

Studies were regarded as qualified for incorporation if they satisfied PICOS requirements outlined below. (i) Population: Patients aged ≥18 years receiving any diagnostic or therapeutic GI endoscopic procedure. (ii) Intervention: intravenous injection of remimazolam procedural sedation. (iii) Comparator: an active control agent, such as propofol or midazolam. (iv) Outcomes: time variables (induction time, sedation time, procedure time and discharge time) as well as adverse events which are categorized by system: respiratory events (apnea, hypoxemia, oxygen desaturation, respiratory depression, and severe coughing); cardiovascular events (hypotension, bradycardia, and tachycardia); injection site pain; and neurological symptoms (headache/dizziness and nausea/vomiting). Additionally, sedation quality was assessed using the deepest sedation depth determined by Modified Observer’s Assessment of Alertness/Sedation (MOAA/S) scale as well as the need for physical patient restraint. (v) Study design: CCTs and RCTs.

### 2.3 Literature screening

After importing all retrieved studies into EndNote 21, we excluded duplicate records. Two independent reviewers (Yuxiao Kong, Anqi Wang) screened all titles and abstracts for potential eligibility. Eligible full-text publications of potentially qualified candidates were independently evaluated against the predefined eligibility standards. Conflicting decisions were addressed through group deliberation or with input from an additional independent investigator. (Wenxia Jia).

### 2.4 Data selection

The following data were obtained from the enrolled trials: basic study details (study title, author list, year of publication, and nation); trial design; demographic data (age, gender, BMI, and American Society of Anesthesiologists (ASA) physical status); patient cohort size; procedure-related features; dose and frequency of sedative regimens, including remimazolam and propofol or midazolam; time-based measures (induction time, sedation time, procedure time, and discharge time); adverse events (apnea, hypoxemia, oxygen desaturation, respiratory depression, and severe coughing, hypotension, bradycardia, and tachycardia, injection site pain, headache/dizziness and nausea/vomiting); deepest level of sedation measured by MOAA/S scale and the restraint of patient.

### 2.5 Methodological quality assessment

Two independent investigators conducted separate assessments of bias risk for all included RCTs employing the RoB 2 instrument (8). This tool appraises bias within the following five separate categories: (i) random assignment procedure, bias, (ii) bias caused by departures from the planned treatment protocols, (iii) bias stemming from incomplete outcome information, (iv) bias involved in measurement and ascertainment of outcomes, and (v) bias present in the reporting of final study findings. Three levels of bias risk (“low,” “some concerns,” or “high”) were applied to each domain. These ratings for separate domains were subsequently combined to aggregate the overall judgment of bias risk for each trial. In the event of conflicting evaluations, an external reviewer was invited to arbitrate and establish a unified conclusion.

### 2.6 Statistical methods

All Statistical evaluations were implemented with Review Manager 5.4. We adopted the Mantel–Haenszel approach to estimate pooled effects for dichotomous variables (e.g., adverse event incidence), with results presented as risk ratios (RR) and 95% CIs. The inverse-variance technique was applied to compute mean differences (MD) with 95% CIs for continuous parameters including time metrics and MOAA/S scores.

To account for potential between-study heterogeneity, the random-effects framework was adopted in all statistical processing. Specifically, we employed the DerSimonian and Laird approach. The extent of heterogeneity was quantified through the Cochran Q test and *I*^2^ index. An *I*^2^ value of 51–100%, 26–50%, and 0–25% to represent high, moderate, and low heterogeneity, respectively. Significant heterogeneity was considered to exist when *p* < 0.10 (Q test) and *I*^2^ > 50%. *A priori* subgroup analyses were carried out to examine potential factors contributing to heterogeneity. Given the substantial between-study heterogeneity, we did not perform additional sensitivity analyses using the fixed-effects model. A two-sided *p*-value of less than 0.05 was used as the standard for achieving statistical significance.

## 3 Results

### 3.1 Search results

From an initial pool of 419 records retrieved by our search (Figure 1), we removed 126 duplicates, leaving 293 for title and abstract screening. We procured complete manuscripts of 23 preliminarily pertinent articles, and after a full-text review, 8 RCTs (9–16) were ultimately incorporated into the analysis.

### 3.2 Description of included studies

Totally, the eight trials enrolled 2,455 patients, of whom 1,349 (54.95%) received remimazolam. Table 1 provided a thorough overview of baseline profiles of the enrolled trials. Cardiovascular events were the most comprehensively reported outcomes: hypotension was documented in all eight studies (2,455 patients) (9–16), and bradycardia was assessed in five studies (1,409 patients, 57.39%) (9, 10, 12, 15, 16). Injection site pain-a local reaction-was evaluated in six studies involving 1,930 patients (78.62%) (11–16). Additionally, induction time (12–14, 16), hypoxemia (10–12, 15), and nausea/vomiting (9, 11, 12, 16) were each assessed in four studies, with data available for 1,364 (55.56%), 1,062 (43.26%), and 779 (31.73%) patients, respectively. The detailed comparative results for procedural timing and safety-related outcomes between remimazolam and control groups are presented in Table 2.

**Table 1:** The primary baseline properties of the enrolled trials.

Study	Design	Publishment year	Sample size, n (E/C)	Mean age (G/C, yr)	Male, n (%)	BMI (G/C, kg m-2)	ASA 1/2, N (%)	Procedure	Experimental group	Control group
Park et al. (9)	Multicenter RCT	2026	64/65	42/39	49(37.98%)	23.1/22.9	100%	upper GI endoscopy	Remimazolam: initial dose of 5 mg; additional 2.5 mg in a minimum of 2 min-interval	Midazolam: initial dose of 2 mg; additional 1 ~ 2 mg in a 2 min-interval
Han et al. (10)	Multicenter RCT	2025	198/198	64.96/64.28	234(58.09%)	23.29/23.04	100%	ERCP	Remimazolam: an initial induction dose of 5 mg	Propofol: an induction dose of 0.5–1 mg/kg
Li et al. (11)	Single-center RCT	2024	83/83	52.7/54.1	83 (50%)	23.5/22.8	100%	GI endoscopy	Remimazolam 0.2 mg/kg	Propofol medium and long-chain fatty emulsion 1.5–2.0 mg/kg > 30 s
Deng et al. (12)	Single-center RCT	2024	50/50	45.68/46.68	67 (67%)	30.58/30.53	72%	colonoscopy	Remimazolam besylate (0.15 mg/kg) plus sufentanil (0.1 μg/kg)	Propofol (2 mg/kg) plus the same dose of sufentanil
Choe et al. (13)	Multicenter RCT	2024	200/200	57.7/55.3	196 (48%)	23.9/23.9	100%	EUS	Remimazolam: an induction dose of 5 mg, followed by intermittent maintenance doses of 2.5 mg	Propofol: an induction dose of 0.5–1 mg/kg followed by intermittent maintenance doses of 10–20 mg
Wang et al. (14)	Multicenter RCT	2022	360/120	44.3/46.4	210 (42%)	22.82/22.87	NA	colonoscopy	Remimazolam besylate 7 mg over 1 min (±5 s)	Propofol 1.5 mg/kg over 1 min (±5 s)
Lu et al. (15)	Multicenter RCT	2022	200/200	70.6/70.1	162 (40.25)	22.2/22.2	99%	upper GI endoscopy	Remimazolam tosilate (300 mg/h) in addition to 50-μg fentanyl	Propofol (3 g/h) in addition to 50-μg fentanyl
Chen et al. (16)	Multicenter RCT	2020	194/190	44.47/44.43	161 (41.93%)	23.19/23.21	100%	colonoscopy	Fentanyl citrate injection + an initial intravenous dose of remimazolam tosylate 5.0 mg	Fentanyl citrate injection + an initial intravenous dose of Propofol 1.5 mg/kg

ERCP, endoscopic retrograde cholangiopancreatography; EUS, endoscopic ultrasound; E, experimental group; C, control group; NA, not available.

**Table 2:** Pooled results for time- and safety-related outcomes.

Outcome measures	Included studies, n	Sample size, n (E/C)	Effect estimate (95% CI)	*P*-value	*I*^2^ (%)
Time variables					
Induction time	4	804/560	MD -0.06 [−0.39, 0.28]	0.74	95
Sedation time	2	264/265	MD -7.92 [−25.17, 9.33]	0.37	99
Procedure time	2	400/400	MD 0.99 [−0.57, 2.56]	0.21	80
Discharge time	2	258/255	MD -7.33 [−22.77, 8.12]	0.35	98
Cardiovascular adverse events					
Hypotension	8	1349/1106	RR 0.59 [0.43, 0.81]	0.001	74
Bradycardia	5	706/703	RR 0.42 [0.25, 0.71]	0.001	5
Tachycardia	3	60/57	RR 1.07 [0.84, 1.35]	0.6	0
Respiratory adverse events					
Apnea	2	248/248	RR 0.51 [0.30, 0.88]	0.01	0
Hypoxemia	4	531/531	RR 0.99 [0.36, 2.75]	0.98	85
Oxygen desaturation	2	560/320	RR 0.20 [0.08, 0.52]	0.0009	0
Respiratory depression	3	598/598	RR 0.45 [0.23, 0.87]	0.02	0
Severe coughing	2	398/398	RR 0.84 [0.24, 2.97]	0.79	0
Neurological symptoms					
Headache/dizziness	3	308/305	RR 0.84 [0.55, 1.28]	0.43	10
Nausea/vomiting	4	391/388	RR 1.27 [0.31, 5.25]	0.74	58
Injection site pain	6	1087/843	RR 0.20 [0.06, 0.66]	0.008	85
Restraint of the patient	2	398/398	RR 0.92 [0.65, 1.29]	0.62	0
Deepest sedation level, MOAA/S score	2	424/185	MD 0.06 [−0.83, 0.94]	0.9	96

CI, confidence interval; MD, mean difference; RR, risk ratio; E, experimental group; C, control group.

### 3.3 Quality assessment

**Figure 2:**
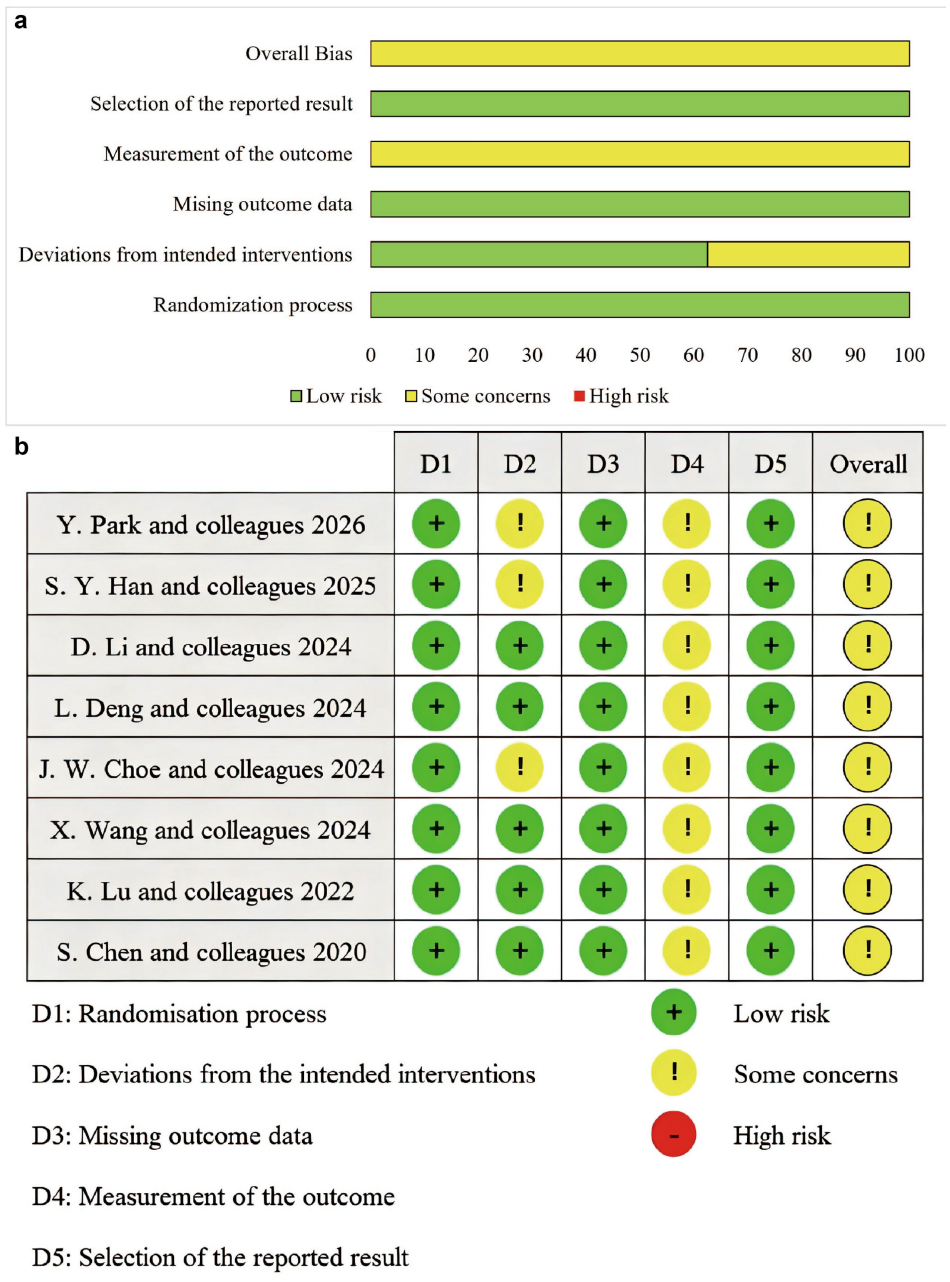
Methodological quality and bias risk evaluation. **(a)** Distribution of bias risk levels. **(b)** Synoptic overview of individual bias risk judgments.

We utilized RoB 2 assessment tool to appraise the potential for bias in the enrolled RCTs, and the resultant data are presented in Figure 2. Three of the eight trials (9, 13, 14) provided insufficient information to assess whether deviations from the intended intervention (e.g., lack of blinding of participants or personnel) might have affected the outcomes, yielding an overall rating of ‘some concerns’ within this domain. All other domains across the included studies were considered as low risk of bias.

### 3.4 Time variables

We defined induction time as the chronological span (minutes) extending from sedative injection to unconsciousness. This outcome was assessed in four studies involving 1,364 participants (12–14, 16). The pooled analysis revealed a MD of −0.06 min (95% CI: −0.38 to 0.28; *p* = 0.74) (Table 2; Supplementary Figure 1a), indicating no clinically or statistically significant difference between the two agents. Although heterogeneity was remarkably high (*I*^2^ = 95%; *p* < 0.00001), the magnitude of the effect was negligible and not in favor of either agent, with moderate between-study variance (*Tau*^2^ = 0.11).

The cumulative sedation interval was calculated as the temporal phase elapsed from the initiation of sedative infusion until the patient regained consciousness or met predefined criteria for adequate awareness and responsiveness (e.g., MOAA/S ≥ 4 or 5). Only two studies (*n* = 529) evaluated sedation duration (9, 15). Patients who received remimazolam had a numerically shorter sedation duration (MD = −7.92 min, 95% CI: −25.17 to 9.33; *p* = 0.37) (Table 2; Supplementary Figure 1b), though this difference was not statistically significant. However, high heterogeneity was observed (*I*^2^ = 99%; Tau^2^ = 153.21; *p* < 0.00001), likely attributable to variations in dosing regimens, patient characteristics, and criteria for defining recovery of consciousness.

Procedure time, defined as the duration from sedation initiation to endoscope removal, was evaluated in two studies (*n* = 800) (13, 15). The aggregated data synthesis uncovered no meaningful intergroup divergence (MD = 0.99 min, 95% CI: −0.57 to 2.56; *p* = 0.21) (Table 2; Supplementary Figure 1c). Moderate heterogeneity was observed (*I*^2^ = 80%; *Tau*^2^ = 1.03; *p* = 0.02), though this did not substantively alter the non-significant effect estimate.

Discharge time, measured from sedative infusion discontinuation to meeting anesthesia discharge criteria, was reported in two studies involving 513 patients (9, 16). Remimazolam showed a trend toward earlier discharge (MD = −7.33 min; 95% CI: −22.77 to 8.12; *p* = 0.35) (Table 2; Supplementary Figure 1d), though this difference did not achieve statistical significance. Substantial heterogeneity was observed (*I*^2^ = 98%; *Tau*^2^ = 122.25; *p* < 0.00001).

### 3.5 Cardiovascular adverse events

**Figure 3:**
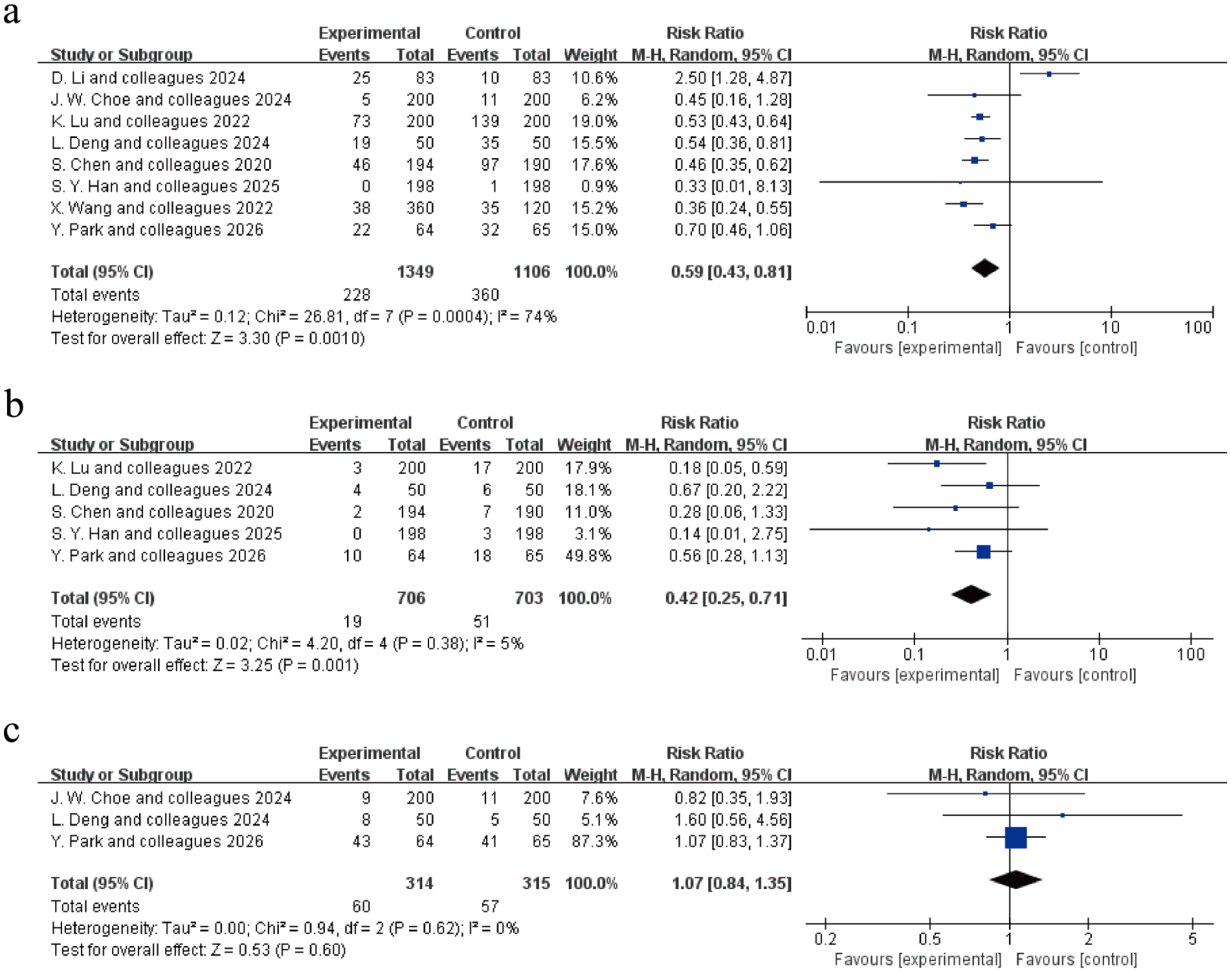
Cardiovascular adverse events analyses. **(a)** Hypotension, **(b)** bradycardia, and **(c)** tachycardia. Remimazolam was linked to considerably decreased rates of hypotension and bradycardia. RR, risk ratio; CI, confidence interval; M-H, Mantel–Haenszel.

The diagnostic criterion for hypotension included a mean arterial pressure (MAP) < 60–65 mmHg, systolic blood pressure (SBP) ≤ 80–90 mmHg, or a ≥ 20% decline in either MAP or SBP compared with baseline values, measured from sedative administration until return of consciousness (17, 18). All eight studies (*n* = 2,455) described the events of hypotension (9–16). Remimazolam was related to a significantly reduced risk of developing hypotension (RR = 0.59; 95% CI: 0.43–0.81; *p* = 0.001) (Table 2; Figure 3a). Moderate heterogeneity was observed (*I*^2^ = 74%; *Tau*^2^ = 0.12; *p* = 0.0004).

Bradycardia was considered present at a heart rate under 50–60 beats per minute. Five studies (*n* = 1,409) evaluated this outcome (9, 10, 12, 15, 16). Remimazolam was linked to a significantly reduced risk of bradycardia (RR = 0.42; 95% CI: 0.25–0.71; *p* = 0.001) (Table 2; Figure 3b). Results were consistent across studies (*I*^2^ = 5%; *Tau*^2^ = 0.02; *p* = 0.38).

We deemed tachycardia as an elevated heart rate exceeding 100 beats per minute. Three studies (*n* = 629) reported tachycardia events (9, 12, 13). The pooled quantitative synthesis showed no obvious distinction when comparing tachycardia incidence (RR = 1.07; 95% CI: 0.84–1.35; *p* = 0.60) (Table 2; Figure 3c). All studies reported similar effect directions and magnitudes, with no detectable heterogeneity (*I*^2^ = 0%; *Tau*^2^ = 0; *p* = 0.62).

### 3.6 Respiratory adverse events

**Figure 4:**
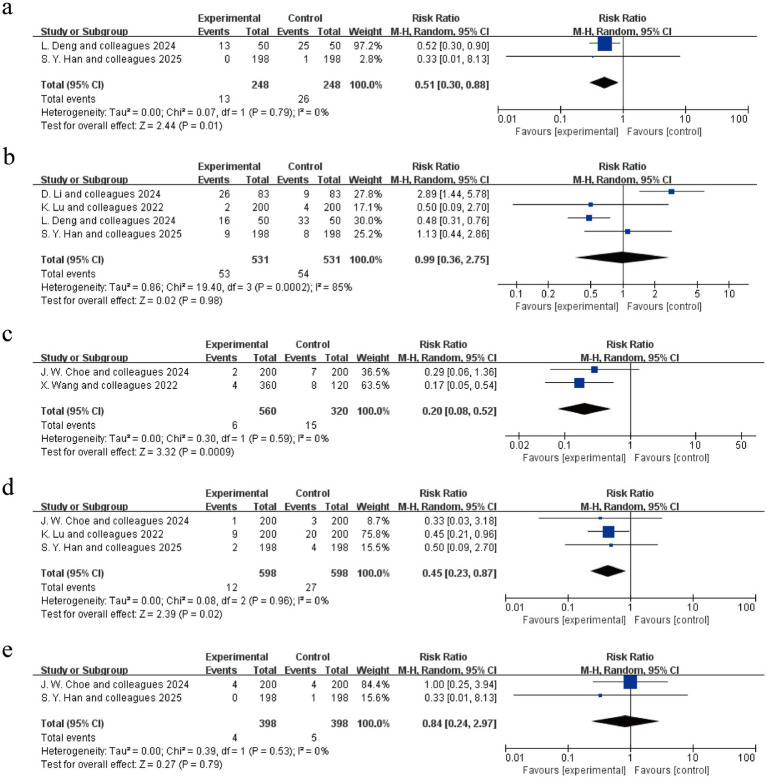
Respiratory adverse events analyses. **(a)** Apnea, **(b)** hypoxemia, **(c)** oxygen desaturation, **(d)** respiratory depression, and **(e)** severe coughing. Remimazolam was linked to a markedly lower risk of respiratory depression. RR, risk ratio; CI, confidence interval; M-H, Mantel–Haenszel.

We defined apnea as the cessation of airflow for at least 10 s during sedation. Two studies (n = 496) reported this outcome (10, 12). Remimazolam conferred a significantly lower probability of apnea (RR = 0.51, 95%CI: 0.30–0.88; *p* = 0.01) (Table 2; Figure 4a). No heterogeneity was observed (*I*^2^ = 0%; *Tau*^2^ = 0.00; *p* = 0.79).

Hypoxemia was assessed as reduced arterial partial pressure of oxygen (PaO_2_), with normal adult values ranging from 83 to 108 mmHg. This definition was strictly adopted from the original trials included in our analysis. Only four studies (*n* = 1,062) using consistent diagnostic criteria for hypoxemia were included (10–12, 15). Despite this strict inclusion, high heterogeneity was still observed (*I*^2^ = 85%; *Tau*^2^ = 0.86; *p* = 0.0002), while pooled data demonstrated no discernible differences between the agents (RR = 0.99; 95% CI: 0.36–2.75; *p* = 0.98) (Table 2; Figure 4b).

Oxygen desaturation was diagnosed if pulse oximetry revealed a peripheral capillary oxygen saturation (SpO₂) of less than 90%. This SpO_2_-based criterion is the most commonly used standard in endoscopic sedation trials. Two studies (*n* = 880) reported this event (13, 14). Remimazolam significantly decreased the rates of oxygen desaturation (RR = 0.2; 95% CI: 0.08–0.52; *p* = 0.0009) (Table 2; Figure 4c). No appreciable heterogeneity was uncovered (*I*^2^ = 0; *Tau*^2^ = 0.00; *p* = 0.59).

Respiratory depression was defined as inadequate alveolar ventilation, diagnosed by a sustained elevation in arterial carbon dioxide partial pressure (PaCO_2_ exceeding 45 mmHg) and/or a persistently reduced respiratory rate (<12 breaths/min), in the absence of primary pulmonary disease. Three studies (*n* = 1,196) evaluated this outcome (10, 13, 15). Patients receiving remimazolam exhibited a 55% decreased risk (RR = 0.45; 95% CI: 0.23–0.87; *p* = 0.02) (Table 2; Figure 4d), with no heterogeneity (*I*^2^ = 0; *Tau*^2^ = 0.00; *p* = 0.96).

Severe coughing was defined as intermittent, intense episodes that either cause complications or severely interfere with speech. Two studies (n = 596) described the events (10, 13). No significant between-group distinction was detected (RR = 0.84; 95% CI: 0.24–2.97; *p* = 0.79) (Table 2; Figure 4e), with no detectable heterogeneity (*I*^2^ = 0%; *Tau*^2^ = 0; *p* = 0.53).

### 3.7 Neurological symptoms

Three studies involving 613 patients reported data on headache, dizziness, or both (9, 12, 16). No statistically significant discrepancy was found in the pooled estimates (RR = 0.84; 95% CI: 0.55–1.28; *p* = 0.43) (Table 2; Supplementary Figure 2a). Heterogeneity was low (*I*^2^ = 10%; *Tau*^2^ = 0.03; *p* = 0.33), indicating consistent results across studies.

Four studies (*n* = 79) reported experiencing nausea and/or vomiting during the postoperative phase (9, 11, 12, 16). The occurrence of these adverse events did not occur much differently between drugs (RR = 1.27; 95% CI: 0.31–5.25; *p* = 0.74) (Table 2; Supplementary Figure 2b) and moderate heterogeneity was observed (*I*^2^ = 58%; *Tau*^2^ = 1.16; *p* = 0.07).

### 3.8 Other indicators

We considered injection site pain as any self-reported or observed discomfort, burning, or tenderness at the venous access site immediately after drug infusion. Six studies (*n* = 1,930) reported data on this outcome (11–16). Remimazolam significantly reduced the likelihood of injection site pain versus the control group (RR = 0.20; 95% CI: 0.06–0.66; *p* = 0.008) (Table 2; Supplementary Figure 3). While heterogeneity was substantial (*I*^2^ = 85%; *Tau*^2^ = 1.59; *p* < 0.00001), the direction of benefit consistently favored remimazolam.

We assessed the deepest level of sedation achieved during procedural sedation using MOAA/S scale, with higher scores indicating lighter sedation (e.g., MOAA/S = 5: fully awake; MOAA/S = 1: unresponsive). Two studies (*n* = 609) reported data on this outcome (9, 14). Between groups, no significant statistical distinction was observed (MD = 0.06; 95% CI: −0.83 to 0.94; *p* = 0.90) (Table 2; Supplementary Figure 4). Substantial heterogeneity was present (*I*^2^ = 96%; *Tau*^2^ = 0.39; *p* < 0.00001).

Two studies (*n* = 596) evaluated the need for patient restraint during sedation as a measure of procedural agitation or inadequate sedation control (10, 13). No evidence of a significant intergroup variation was found (RR = 0.92; 95% CI: 0.65–1.29; *p* = 0.62) (Table 2; Supplementary Figure 5). Heterogeneity was absent (*I*^2^ = 0%; *Tau*^2^ = 0; *p* = 0.60), indicating consistent results across studies. Both drugs demonstrated similar efficacy in maintaining adequate sedation without requiring physical restraint.

## 4 Discussion

The present meta-analysis comprised eight RCTs with 2,455 adult patients (1,349 remimazolam, 1,106 control) who underwent gastrointestinal endoscopic procedures. Our findings indicate that, compared with conventional sedative agents, remimazolam demonstrates significant advantages in safety-related outcomes, particularly cardiovascular and respiratory adverse events, while showing non-inferiority in procedural time-related metrics and sedation quality.

Remimazolam, a novel benzodiazepine derivative, has received regulatory approval for clinical use in inducing and maintaining general anesthesia in China, South Korea, and Japan, as well as for procedural sedation in China, US, UK, and EU (6, 19, 20). In Belgium, it is also authorized as an adjunctive sedative for mechanically ventilated ICU patients with COVID-19 (21). As a selective GABA_A_ receptor agonist, remimazolam provides rapid-onset sedation with a predictable and controllable duration, and its effects are readily reversible with flumazenil (22). However, to support its broader and more informed clinical use, further clinical evidence is required to fully establish its effectiveness and safety profile in procedural sedation.

Our findings align with existing clinical evidence underscoring remimazolam’s favorable safety profile, particularly its decreased risk of hypotension, bradycardia, apnea, hypoxemia, respiratory compromise, and pain at the site of drug administration during sedation. This may be attributed to its selective agonism at GABA_A_ receptors and rapid metabolism by tissue esterases, thus together result in minimal cardiovascular and respiratory depressive effects (22). Numerous clinical trials have shown that remimazolam is linked to noticeably lower rates of cardiovascular adverse events and injection pain, consistent with our results (hypotension: RR = 0.59; bradycardia: RR = 0.42; injection pain: RR = 0.20) (23–25). Nevertheless, there remains a lack of direct clinical evidence conclusively demonstrating its superior respiratory maintenance capability. While indirect indicators such as lower rates of apnea and hypoxemia suggest better respiratory stability (apnea: RR = 0.51; hypoxemia: RR = 0.20; respiratory depression: RR = 0.45), further studies using objective respiratory parameters (e.g., minute ventilation, end-tidal CO_2_, or continuous oxygen saturation monitoring) are needed to validate this potential benefit.

Substantial heterogeneity was observed for several key outcomes in our meta-analysis. The potential sources of this heterogeneity are multifactorial and are discussed below to aid appropriate interpretation of pooled estimates. First, divergent dosing regimens of remimazolam and control agents represent a major source of heterogeneity. Variations in initial and maintenance doses, as well as fixed versus weight-based dosing strategies, could lead to differences in sedation efficacy and time-related outcomes, including induction time and sedation time. Second, heterogeneity may also arise from variations in baseline patient characteristics. Differences in age, body mass index, and comorbidities - even among ASA I - II patients - can alter responses to sedatives and affect pooled results. Third, the type and complexity of GI endoscopic procedures varied considerably across studies, ranging from simple diagnostic endoscopies to more complex therapeutic procedures such as ERCP. These differences may entail distinct sedation requirements and influence outcomes including discharge time. Despite the above exploration of potential heterogeneity sources, we were unable to perform further subgroup and sensitivity analyses as suggested. With only eight eligible trials included, stratification by comparator agent, endoscopic type, concomitant opioids, age and dose regimen would lead to insufficient sample size and unreliable statistical results. For this reason, all pooled results should be interpreted prudently.

This meta-analysis possesses some notable advantages, chief among them being the exclusive inclusion of RCTs only, which substantially reduces the risk of confounding commonly encountered in observational studies. In addition, the meta-analysis encompasses a substantial total sample size and adheres to rigorous methodology as prespecified in our protocol.

Although previous systematic reviews have evaluated remimazolam in procedural sedation, this meta-analysis provides a more focused comparison against both midazolam and propofol across multiple clinically relevant outcomes-including cardiovascular and respiratory adverse events, time to neurological symptoms. By exclusively including RCTs and applying rigorous methodology, our findings offer updated, quantitatively pooled evidence supporting remimazolam’s potential advantages in safety and patient comfort. Importantly, we explicitly address the influence of concomitant sedative-analgesic use and the limitations imposed by the still-limited number of available RCTs, thereby providing a balanced, up-to-date synthesis to inform future clinical and research directions.

Based on current evidence, remimazolam may be considered as an alternative to midazolam or propofol for intravenous sedation during low- to moderate-risk GI endoscopic procedures, particularly in patients in whom a reduced incidence of respiratory or cardiovascular depression and rapid, clear-headed recovery are desired. Its sedative effect can be rapidly reversed by flumazenil, which may be advantageous in settings where oversedation is a concern. However, because sedation is typically achieved using a combination of sedative and analgesic agents, the choice of remimazolam should take into account the type and dose of co-administered opioids. Until further large-scale RCTs are available, its use for general anesthesia or in high-risk (ASA III-IV) populations should be guided by local protocols and clinician judgment.

However, several limitations should be acknowledged. Firstly, this meta-analysis included only eight randomized controlled trials, which may reduce the generalizability and robustness of our pooled results. Therefore, more large-sample, well-designed trials are required to verify our conclusions. Secondly, only English-language papers were included in the search, which may entail a susceptibility to selection bias and restrict the breadth of the evidential foundation. Thirdly, the review protocol was prospectively registered on PROSPERO after we completed literature retrieval but prior to the commencement of formal literature screening, data extraction and statistical analysis. This timeline complies with the optimal methodological standard for systematic reviews, which requires protocol registration before initiating screening procedures. Fourthly, the majority of included trials recruited individuals graded as ASA physical status I or II; only one trial incorporated 28 participants with ASA status III (12). As a result, the applicability of our results to high-risk populations, including elderly patients with multiple comorbidities and those undergoing advanced endoscopic interventions, is significantly limited. Fifthly, most studies did not comprehensively report time-related outcomes, which rendered our planned meta-analysis of the full set of time metrics unfeasible. Sixthly, outcome definitions were finalized after data extraction grounded in the most prevalent evaluative criteria across the analyzed trials; however, several trials provided insufficient detail to fully align these definitions. Seventhly, gastrointestinal endoscopic sedation typically requires combined sedative and analgesic regimens. The inconsistent types, dosages, and administration protocols of concomitant sedatives and opioids across trials may interact with remimazolam and confound its efficacy and safety outcomes. Eighthly, seven of the eight included trials adopted propofol as the comparator, while only one trial used midazolam. Due to the extremely small number of midazolam-controlled studies, subgroup analysis stratified by comparator type was statistically unfeasible. Pooling all control groups together may mask potential clinical differences between different conventional sedatives, and this imbalance should be noted when interpreting our findings. In addition, incomplete reporting of vasopressor use further interferes with the accurate interpretation of cardiovascular adverse events. Ninthly, considerable between-study heterogeneity was observed for certain outcomes. Tenthly, given that fewer than 10 studies were included for all outcomes, formal assessment of publication bias via funnel plots or statistical tests could not be performed reliably. The potential impact of unpublished negative studies on our pooled results therefore cannot be ruled out.

## 5 Conclusion

Our findings from this meta-analysis of eight RCTs suggest that remimazolam may reduce the incidence of cardiovascular and respiratory adverse events as well as injection-related pain compared with midazolam and propofol. These potential advantages in safety and patient comfort support its promising role in gastrointestinal procedural sedation. However, these findings should be interpreted with caution due to substantial heterogeneity for several outcomes and the limited number of included trials. Moreover, most participants were classified as ASA I or II, restricting the generalizability to high-risk populations. Therefore, further large-scale, high-quality randomized controlled trials are warranted to confirm these observations and to better define the role of remimazolam in clinical practice.
