# Supplementary material for: Efficacy and safety of remimazolam for sedation in gastrointestinal endoscopy: a systematic review and meta-analysis
Source: Front Med (Lausanne). 2026 Jun 24;13:1811767. doi: 10.3389/fmed.2026.1811767 (PMC13342045; doi:10.3389/fmed.2026.1811767)
Supplement: Supplementary file 1 [file Table_1.DOCX]

Supplementary Material

## Supplementary Tables

**Supplementary Table 1.** Search summary

| Topic | Remimazolam in gastrointestinal endoscopy |
| --- | --- |
| Reference manager | Endnote 21 |
| Restrictions or limitations | Controlled Clinical Trial, Randomized Controlled Trial |
| Databases | PubMed, Embase, Cochrane |
| Date run | 31/1/2026 |
| Total number of results | 419 |
| Search prepared by | Yuxiao Kong, Anqi Wang |

# **PubMed**

| **Search number** | **Query** | **Filters** | **Results** | **Date** |
| --- | --- | --- | --- | --- |
| 6 | (((remimazolam[Title/Abstract]) OR (CNS-7056[Title/Abstract])) OR (Byfavo[Title/Abstract])) AND (("Endoscopy, Gastrointestinal"[Mesh]) OR (((((Endoscopic Gastrointestinal Surgery[Title/Abstract]) OR (Gastrointestinal Endoscopic Surgical Procedures[Title/Abstract])) OR (Procedure*, Endoscopic Gastrointestinal, Surgical[Title/Abstract])) OR (Procedure*, Gastrointestinal Endoscopic Surgical[Title/Abstract])) OR (Surgical Procedure*, Endoscopic Gastrointestinal[Title/Abstract]))) | Controlled Clinical Trial, Randomized Controlled Trial | 48 | 2026/1/31 |
| 5 | (((remimazolam[Title/Abstract]) OR (CNS-7056[Title/Abstract])) OR (Byfavo[Title/Abstract])) AND (("Endoscopy, Gastrointestinal"[Mesh]) OR (((((Endoscopic Gastrointestinal Surgery[Title/Abstract]) OR (Gastrointestinal Endoscopic Surgical Procedures[Title/Abstract])) OR (Procedure*, Endoscopic Gastrointestinal, Surgical[Title/Abstract])) OR (Procedure*, Gastrointestinal Endoscopic Surgical[Title/Abstract])) OR (Surgical Procedure*, Endoscopic Gastrointestinal[Title/Abstract]))) |  | 74 | 2026/1/31 |
| 4 | ("Endoscopy, Gastrointestinal"[Mesh]) OR (((((Endoscopic Gastrointestinal Surgery[Title/Abstract]) OR (Gastrointestinal Endoscopic Surgical Procedures[Title/Abstract])) OR (Procedure*, Endoscopic Gastrointestinal, Surgical[Title/Abstract])) OR (Procedure*, Gastrointestinal Endoscopic Surgical[Title/Abstract])) OR (Surgical Procedure*, Endoscopic Gastrointestinal[Title/Abstract])) |  | 108,044 | 2026/1/31 |
| 3 | ((((Endoscopic Gastrointestinal Surgery[Title/Abstract]) OR (Gastrointestinal Endoscopic Surgical Procedures[Title/Abstract])) OR (Procedure*, Endoscopic Gastrointestinal, Surgical[Title/Abstract])) OR (Procedure*, Gastrointestinal Endoscopic Surgical[Title/Abstract])) OR (Surgical Procedure*, Endoscopic Gastrointestinal[Title/Abstract]) |  | 68 | 2026/1/31 |
| 2 | "Endoscopy, Gastrointestinal"[Mesh] |  | 108,002 | 2026/1/31 |
| 1 | ((remimazolam[Title/Abstract]) OR (CNS-7056[Title/Abstract])) OR (Byfavo[Title/Abstract]) |  | 1,199 | 2026/1/31 |

**Embase**

| **No.** | **Query** | **Results** | **Date** |
| --- | --- | --- | --- |
| #7 | #3 AND #6 | 248 | 31-Jan-26 |
| #6 | #4 OR #5 | 231,373 | 31-Jan-26 |
| #5 | 'endoscopy, gastrointestinal':ti,ab,kw OR 'gastrointestinal endoscopy':ti,ab,kw | 19,628 | 31-Jan-26 |
| #4 | 'gastrointestinal endoscopy'/exp | 226,423 | 31-Jan-26 |
| #3 | #1 OR #2 | 1,713 | 31-Jan-26 |
| #2 | 'anerem':ti,ab,kw OR 'biprazine pf':ti,ab,kw OR 'byfavo':ti,ab,kw OR cns*7056*:ti,ab,kw OR gw*502056*:ti,ab,kw OR hr*7056*:ti,ab,kw OR ono*2745*:ti,ab,kw OR ono*in251*:ti,ab,kw OR rf*10007*:ti,ab,kw OR sp*148.5*:ti,ab,kw OR '3-[8-bromo-1-methyl-6-(2-pyridinyl)-4h-imidazo[1,2-a][1,4]benzodiazepin-4-yl]propanoic acid methyl ester':ti,ab,kw OR '3-[8-bromo-1-methyl-6-(pyridin-2-yl)-4h-imidazo[1,2-a][1,4]benzodiazepin-4-yl]propanoic acid methyl ester':ti,ab,kw OR '8-bromo-1-methyl-6-(2-pyridinyl)-4h-imidazo[1,2-a][1,4]benzodiazepine-4-carboxylic acid methyl ester':ti,ab,kw OR '8-bromo-1-methyl-6-(pyridin-2-yl)-4h-imidazo[1,2-a][1,4]benzodiazepine-4-carboxylic acid methyl ester':ti,ab,kw OR 'methyl 3-[8-bromo-1-methyl-6-(2-pyridinyl)-4h-imidazo[1,2-a][1,4]benzodiazepin-4-yl]propanoate':ti,ab,kw OR 'methyl 3-[8-bromo-1-methyl-6-(pyridin-2-yl)-4h-imidazo[1,2-a][1,4]benzodiazepin-4-yl]propanoate':ti,ab,kw | 48 | 31-Jan-26 |
| #1 | 'remimazolam'/exp | 1,710 | 31-Jan-26 |

# **Cochrane**

Search Name: Remimazolam in gastrointestinal endoscopy

Date Run: 31/01/2026 11:38:12

Comment:

| **ID** | **Search** | **Hits** |
| --- | --- | --- |
| #1 | MeSH descriptor: [Endoscopy, Gastrointestinal] explode all trees | 6,606 |
| #2 | ((Endoscopy, Gastrointestinal) OR (Endoscopic Gastrointestinal Surgery) OR (Gastrointestinal Endoscopic Surgical Procedures) OR (Procedure*, Endoscopic Gastrointestinal, Surgical) OR (Procedure*, Gastrointestinal Endoscopic Surgical) OR (Surgical Procedure*, Endoscopic Gastrointestinal)):ti,ab,kw | 6,128 |
| #3 | ((Remimazolam) OR (CNS-7056) OR (Byfavo)):ti,ab,kw | 1,544 |
| #4 | #1 OR #2 | 11,041 |
| #5 | #3 AND #4 in Trials | 123 |
