# Supplementary material for: Efficacy and safety of remimazolam for sedation in gastrointestinal endoscopy: a systematic review and meta-analysis
Source: Front Med (Lausanne). 2026 Jun 24;13:1811767. doi: 10.3389/fmed.2026.1811767 (PMC13342045; doi:10.3389/fmed.2026.1811767)
Supplement: Supplementary file 2 [file Table_2.DOCX]

Supplementary Material

## Supplementary Figures


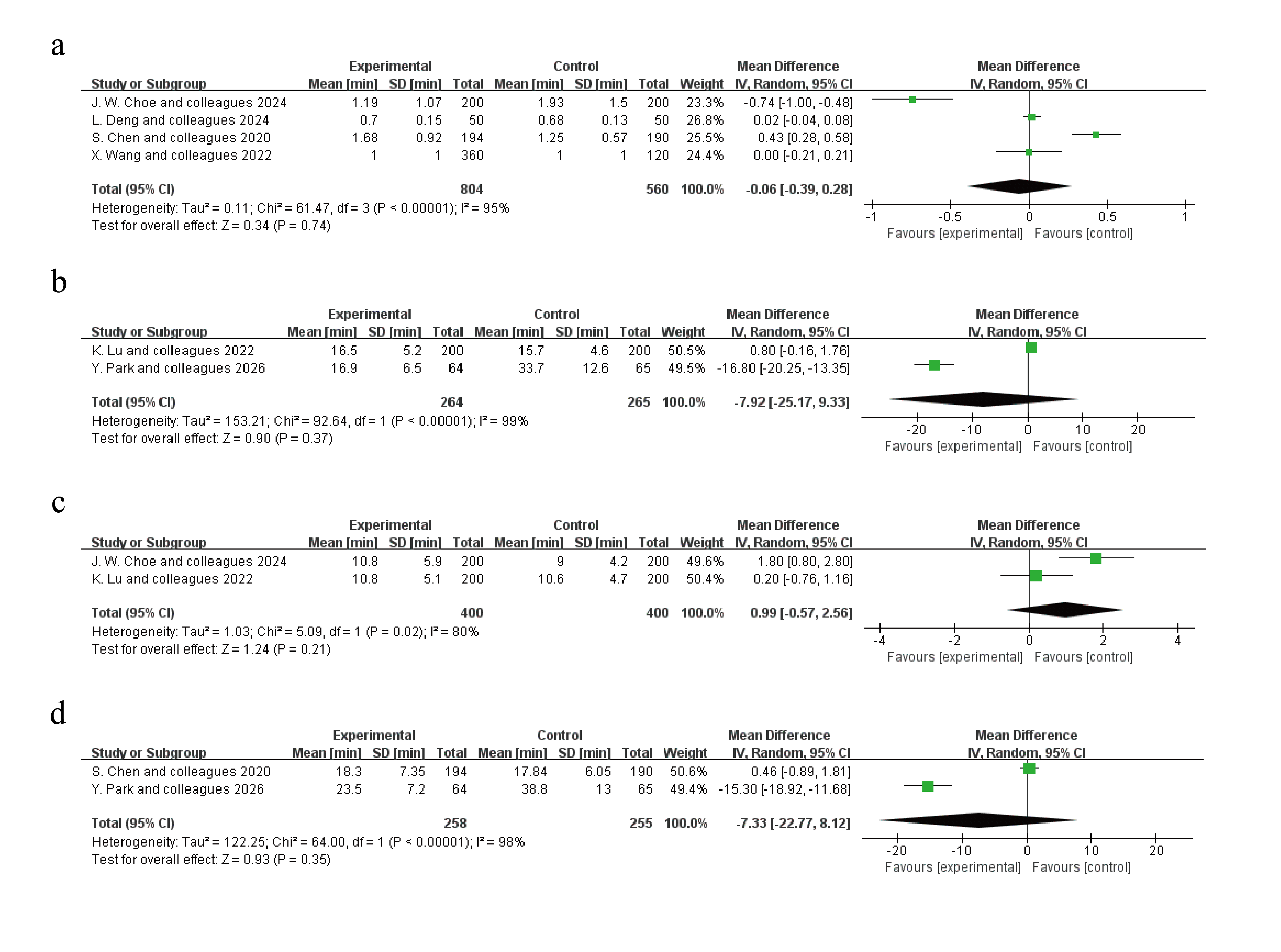


**Supplementary Figure 1.** Forest plots comparing the following time variables between the two groups: a, induction time; b, sedation time; c, procedure time; and d, discharge time. CI, confidence interval; IV, inverse-variance; MD, mean difference; SD, standard deviation.


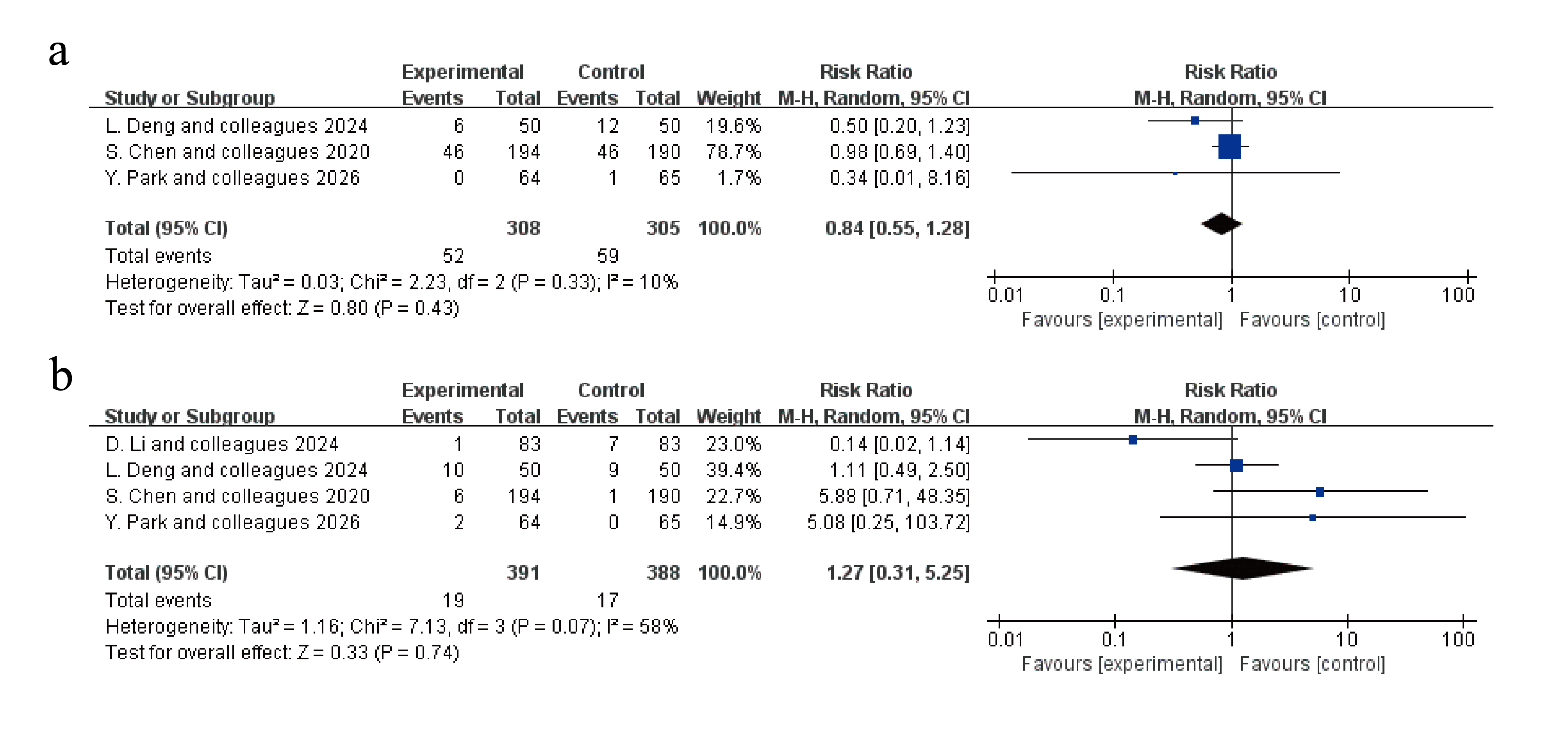


**Supplementary Figure 2.** Forest plots comparing the following neurological symptoms between the two groups: a, headache and dizziness; b, nausea and vomiting. No difference was observed between the sedative drugs. RR, risk ratio; CI, confidence interval; M-H, Mantel-Haenszel.


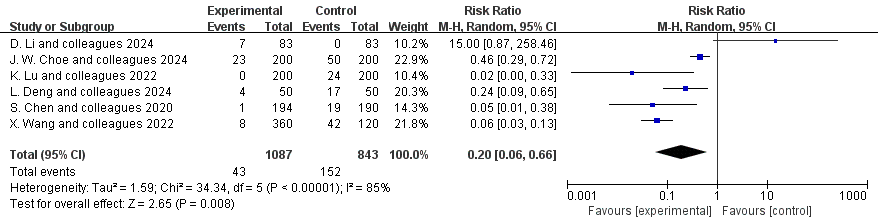


**Supplementary Figure 3.** Forest plot comparing the injection site pain between the two groups. The remimazolam group significantly reduced the incidence of injection site pain (RR = 0.20, 95% CI: 0.06–0.66, P = 0.008). RR, risk ratio; CI, confidence interval; M-H, Mantel-Haenszel.


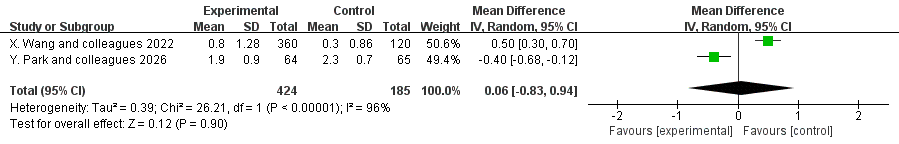


**Supplementary Figure 4.** Forest plot comparing the deepest sedation level, MOAAS score between the two groups. No difference was observed between the sedative drugs. CI, confidence interval; IV, inverse-variance; MD, mean difference; SD, standard deviation.


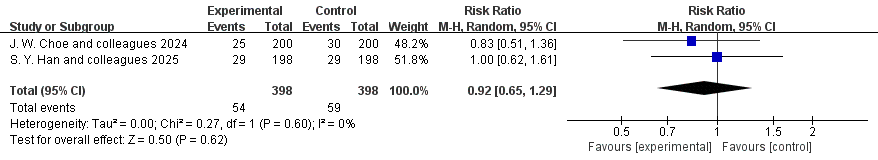


**Supplementary Figure 5.** Forest plot comparing the restraint of the patient between the two groups. No difference was observed between the sedative drugs. RR, risk ratio; CI, confidence interval; M-H, Mantel-Haenszel.
